# Substrate reduction therapy for inborn errors of metabolism

**DOI:** 10.1042/ETLS20180058

**Published:** 2019-02-08

**Authors:** Wyatt W. Yue, Sabrina Mackinnon, Gustavo A. Bezerra

**Affiliations:** Structural Genomics Consortium, Nuffield Department of Clinical Medicine, University of Oxford, Oxford OX3 7DQ, U.K.

**Keywords:** drug discovery and design, gene silencing, inborn errors of metabolism, small molecules, substrate reduction

## Abstract

Inborn errors of metabolism (IEM) represent a growing group of monogenic disorders each associated with inherited defects in a metabolic enzyme or regulatory protein, leading to biochemical abnormalities arising from a metabolic block. Despite the well-established genetic linkage, pathophysiology and clinical manifestations for many IEMs, there remains a lack of transformative therapy. The available treatment and management options for a few IEMs are often ineffective or expensive, incurring a significant burden to individual, family, and society. The lack of IEM therapies, in large part, relates to the conceptual challenge that IEMs are loss-of-function defects arising from the defective enzyme, rendering pharmacologic rescue difficult. An emerging approach that holds promise and is the subject of a flurry of pre-/clinical applications, is substrate reduction therapy (SRT). SRT addresses a common IEM phenotype associated with toxic accumulation of substrate from the defective enzyme, by inhibiting the formation of the substrate instead of directly repairing the defective enzyme. This minireview will summarize recent highlights towards the development of emerging SRT, with focussed attention towards repurposing of currently approved drugs, approaches to validate novel targets and screen for hit molecules, as well as emerging advances in gene silencing as a therapeutic modality.

## Introduction

### The premise and promise of substrate reduction therapy

The term inborn errors of metabolism (IEM), first coined by Sir Archibald Garrod in 1908 [1], describes a diverse group of genetic disorders in which a defect in a particular enzyme, transporter, or regulatory protein, results in a malfunctioning metabolic pathway [2]. The biochemical consequences include toxic accumulation of metabolites preceding the defective enzymatic step in the pathway, abnormal intermediates arising from diversion of metabolites into alternative pathways, and deficiency of essential products normally generated downstream of the defect. IEMs are often considered as loss-of-function (LOF) disorders [3], due to the resulting under-physiological level of the defective protein, and associated biochemical abnormalities. A recent nosology study has defined as many as 1015 individual IEM disorders [4], noting that the underlying causes of disease remain undetermined for at least half of them [5]. While individually rare, IEMs collectively are common diseases with an overall incidence reported between 1 in 800 [6] to 2500 [7] live births, thereby contributing to substantial childhood morbidity and mortality worldwide.

Despite the advances in uncovering the genetic, biochemical, and pathological mechanisms for IEMs, the number of approved therapies remains very small (circa 70–80). This gap is partly attributable to the conceptual challenge underlying the drug development for LOF disorders. The intuitive therapeutic target for IEMs, namely the defective enzyme harbouring a LOF mutation *per se*, would imply a strategic imperative to repair or mitigate the deficient or defective enzyme. This avenue is much less travelled in the drug development industry, which has instead more traction in tackling diseases with gain-of-function pathologies via pharmacologic down-regulation of DNA, mRNA, and protein levels [8]. It is therefore no surprise that the search for potential IEM treatment covers a broader and more diverse array of therapeutic modalities ranging from enzyme replacement, gene therapy, and cell/organ transplantation, to small molecule therapies such as pharmacological chaperoning (PC) ([9,10] for review). Nevertheless, IEMs often present as neurological disorders with CNS damage that is not being addressed by current treatments. As such, small molecule therapies that potentially cross the blood–brain barrier (BBB) hold great promise (see [11] for review).

To maximize intervention possibilities, there is rationale to survey the various steps surrounding the defective enzyme in a metabolic pathway, in search for possible therapeutic targets. Pertinent to this, the concept of substrate reduction therapy (SRT) has recently gained wide interest [12]. Rather than directly correcting the enzyme defect, SRT aims to attenuate the bioavailability of the compound that cannot be fully metabolized by the defective enzyme (‘substrate reduction’), thereby restoring a steady-state balance of the pathway to lower the burden of the accumulating substrate. Dietary treatment of phenylketonuria (PKU) is often considered the first original application of SRT, albeit not a pharmaceutical. Here, a phenylalanine (Phe)-restricted diet has been administered for over five decades [13], to regulate the high plasma Phe level in PKU patients caused by defective phenylalanine hydroxylase (PAH), the Phe-metabolizing enzyme.

Nowadays, SRT has gained wide appeal because of the possible administration using small molecule inhibitors that can be taken orally, cross the BBB to address CNS pathology, and avoid immune response, thus overcoming some of the limitations from existing treatments [11]. SRT would particularly apply to the intoxification type of IEMs where toxic metabolite accumulation leads to acute clinical decompensation. SRT would also benefit those IEMs where the accumulated metabolite is a complex polymer that is inappropriately stored within cells over time due to the enzyme defect [e.g. glycogen storage disorders (GSDs), lysosomal storage disorders (LSDs)].

## Main body

### Two classical SRT examples and their novel repurposing applications

**Figure 1.**
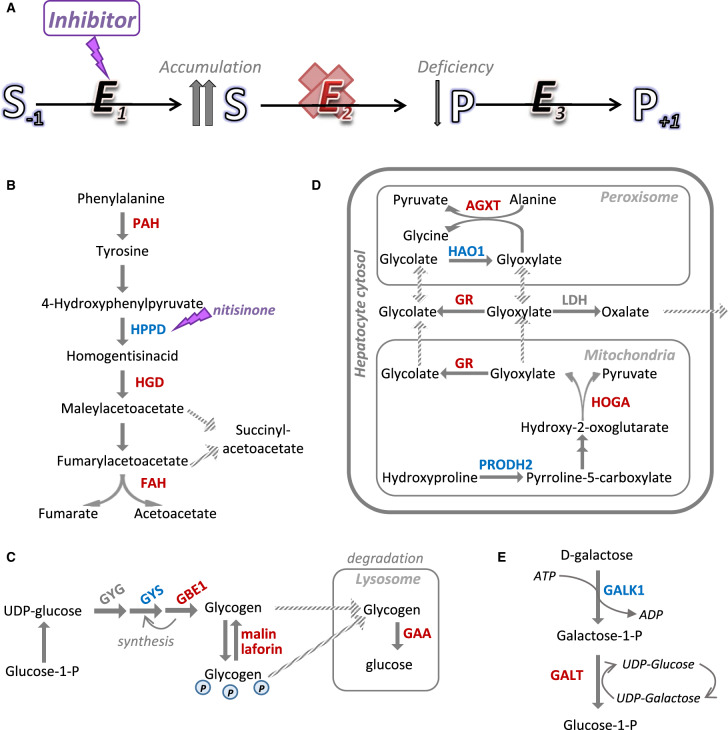
SRT examples applied to linear pathways of metabolite interconversion. (**A**) Schematic diagram illustrating the general concept of SRT, where E_2_ is the defective enzyme, and E_1_ is the inhibitory target for SRT. (**B**) Pathway of tyrosine degradation. (**C**) Pathway of glycogen biosynthesis and targeted degradation in lysosome. (**D**) Pathway of glyoxylate metabolism in hepatocytes. (**E**) Pathway of galactose metabolism. For (**B**–**E**), blue letters denote enzymes targeted by SRT, red letters denote the defective enzymes.

For the purpose of this minireview, we broadly categorize the current pre-clinical and clinical applications of SRT into two types. One type applies to metabolic defects arising from an enzyme that is situated within a sequence of reaction steps, often with linear directionality (Figure 1A). Here, the rationale for SRT is to target an upstream enzyme in the pathway, to inhibit biosynthesis of the metabolite that accumulates (to a toxic level) due to a downstream deficient enzyme. A classic example is nitisinone (Orfadin^TM^, Sobi) for the treatment of hereditary tyrosinaemia type 1 (HT-1, OMIM 276700) [14], an IEM caused by deficiency of fumarylacetoacetate hydrolase (FAH) which catalyses the sixth enzymatic step of tyrosine degradation (Figure 1B). The pathogenic mechanism of HT-1 is proposed to be the toxicity of succinylacetone in the liver and kidney arising from the accumulation of FAH substrate, fumarylacetoacetate. Nitisinone, a triketone compound originally developed as an agrochemical [15], has now been used for over two decades for the treatment of HT-1. Its mode of action is by inhibiting 4-hydroxyphenylpyruvate dioxygenase (HPPD), the third enzyme of the tyrosine degradation pathway.

The efficacy of nitisinone has also been assessed for another defect of the same pathway, namely alkaptonuria (MIM 203500), caused by mutations of homogentisate dioxygenase (HGD, fourth step) (Figure 1B). Nitisinone decreased accumulation of homogentisic acid, the toxic precursor of an ochronotic pigment [16]. In an observational study [17], nitisinone was offered off-label to alkaptonuria patients, which slowed down clinical progression [18]. A non-classical application of nitisinone also worthy of mention is the pharmacological inhibition of tyrosine degradation in a mouse model of PKU. This compensates for the deficiency of brain l-tyrosine that is essential for dopamine synthesis, due to the defective enzyme in PKU, namely PAH which converts l-phenylalanine to l-tyrosine [19] (Figure 1B).

**Figure 2.**
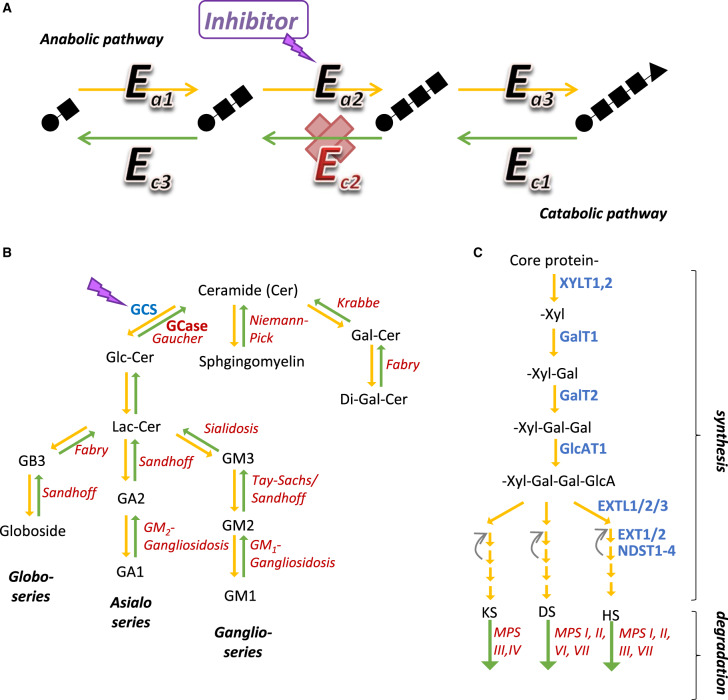
SRT examples applied to biosynthesis and degradation pathway of a storage material. (**A**) Schematic diagram illustrating the general concept of SRT, where *E_a1_*, *E_a2_*_,_ and *E_a3_* are anabolic enzymes for biosynthesis (yellow arrows) of storage material, while *E_c1_*, *E_c2_*_,_ and *E_c3_* are catabolic enzymes for degradation (green arrows). In this scheme, the defective enzyme is *E_c2_*, while the target enzyme for SRT is *E_a2_*. (**B**) Pathway of sphingolipid biosynthesis (yellow arrows) and degradation (green arrows). (**C**) Pathway of glycosaminoglycan biosynthesis (yellow arrows) and degradation (green arrows). For (**B**,**C**), blue letters denote enzymes proposed as SRT targets, to address the metabolic defects shown in red letters.

Another type of SRTs can be illustrated from their application towards LSDs. They represent a heterogeneous group of metabolic defects in lysosomal hydrolases, which are enzymes responsible for the degradation of macromolecular lipids and carbohydrates. Biochemically, LSDs are characterized by the toxic accumulation of intermediates as storage materials in various tissues and organs. Norman Radin [20] first proposed SRT in the 1980s as an approach to compensate for the impaired degradation of storage material due to a defective hydrolase, by inhibiting the corresponding series of enzymes that catalyse the synthesis of the storage material, in order to correct the imbalance between synthesis and degradation (Figure 2A).

The classic SRT example for LSDs is found in the metabolism of glycosphingolipids (GSL), whereby their synthesis involves stepwise transfers of monosaccharide units onto ceramide by a set of glycosyltransferases in the Golgi apparatus, while their breakdown in the lysosome involves an entirely different set of hydrolase enzymes (Figure 2B). The first SRT drug that received market approval is the *N*-alkylated iminosugar Miglustat (*N*-butyldeoxynojirimycin, traded as Zavesca™, Actelion) ([21] for review). Miglustat inhibits glucosylceramide synthase (GCS, or UDP-glucose:ceramide glucosyltransferase), the first enzyme in the GSL biosynthesis pathway, in order to reduce synthesis of glucosylceramide that accumulates in Gaucher's disease (GD, MIM 230800), a disorder of the defective hydrolase β-glucosylceramidase (GCase or GBA1) which is essential for sphingolipid degradation. Miglustat had previously been evaluated clinically as an anti-viral agent because of its inhibition towards viral α-glucosidase I/II as a glucose mimetic. Since 2003, it has been approved for the treatment of non-neuropathic (type 1) GD, as the second-line therapy for adults who are unsuitable for enzyme replacement therapy (ERT). Emerging evidence points to the therapeutic potential of miglustat for type 1 GD being partly attributable to its off-target inhibitory effects, such as towards non-lysosomal β-glucosidase 2 (GBA2) [22,23], suggesting that substrate reduction may not be the sole mode of action.

More recently eliglustat (Cerdelga™, Sanofi Genzyme), a more potent highly-specific ceramide-mimetic inhibitor, with less adverse side effects than miglustat [24] and similar efficacy to enzyme replacement [25–27], was licenced as the first-line therapy in 2014 [28,29], for GD1 adults with suitable CYP2D6 metabolizing status. Nevertheless, effective treatment of the neuropathic GD types 2 (MIM 230900) and 3 (MIM 231000) remains challenging, as eligustat does not cross the BBB as this molecule is recognized by the multidrug resistance protein MDR1 [30]. Newer series of CNS-accessible inhibitors (e.g. Lucerastat, Ibiglustat/Venglustat) have been recently reported to show promising pre-clinical and pharmacokinetic results for GD type 2 [31–33].

Many studies are building onto the premise that a single oral drug could have therapeutic potentials for multiple LSDs, particularly those with neuropathic manifestations. Because glucosylceramide is the common precursor for the various GSL series, it is no surprise that miglustat, eliglustat, and other GCS inhibitors have been tested for storage disorders of ganglio-series GSL (e.g. Tay-Sachs, MIM 272800 [34]; Sandhoff, MIM 268800 [35]; GM1-gangliosidosis, MIM 230500 [36]) and of globo-series GSL (Fabry, MIM 301500 [33,37]) (Figure 2B).

Miglustat has also been approved for the treatment of Niemann–Pick disease type C (NPC; MIM 257220), a disorder of disrupted intracellular lipid trafficking to lysosome that leads to neuronal accumulation of ganglioside GSL (Figure 2B), hence exhibiting similar pathology to GD. Miglustat was shown to slow down NPC disease progression [38], and is now considered as a disease-modifying therapy that could extend patients' life. Another study, however, did not show stabilization of the neurological decline [39]. For further clinical studies, newer more potent iminosugar-based inhibitors are in the pre-clinical development pipeline [40].

### Proof-of-concept for the next-generation SRT targets

The example of miglustat neatly illustrates how repurposing approved drugs to novel indications, incentivized by licensing and marketing provisions from the Orphan Drug Act in 1983 [41,42], can open up new therapeutic opportunities for other IEMs with related pathology. Yet for IEMs without suitable repurposing options, the identification of novel targets and new molecular entity drugs remains a clear priority. For this, proof-of-concept (POC) studies in patient-derived cells or organism models of disease, aimed at demonstrating phenotypic rescue of phenotype and lack of toxicity by the novel therapeutic modality in question, are highly desirable assets before investment of resources into drug development. The POC can involve inhibition studies using existing tool compounds known for the enzyme or the target's biological pathway. Genetic manipulation using small interference RNA (siRNA) and antisense oligonucleotide (ASO) technologies are also emerging as tools to validate substrate reduction mechanism. Furthermore, reports from naturally occurring human variants of the target could inform whether modulation of its mRNA and protein level is viable. Here, we describe recent POC examples targeting intoxification-type IEMs caused by three different types of storage materials.

#### Glycosaminoglycan

Like GSL, the metabolism of glycosaminoglycans (GAGs), which are unbranched disaccharide-based polymers attached to proteins at cell surface or in extracellular matrix, requires distinct stepwise reactions for their biosynthesis and degradation (Figure 2C). Inherited defects in various GAG degradation steps [particularly for dermatan sulfate, heparan sulfate (HS), and keratan sulfate] are the molecular cause of mucopolysaccharidoses (MPS, of seven clinical subtypes), where the neuropathology arises from accumulation of undegraded intermediates in the brain and as such MPS patients are not responsive to current approved ERT treatments for several subtypes (for review, see [43]). There is rationale to target GAG biosynthesis by the SRT approach. Early pre-clinical studies focussed on genistein [44], a soybean isoflavone that inhibits tyrosine kinase activity of EGF-mediated signal transduction, to silence the gene expression of GAG biosynthetic enzymes. Clinical trials for MPS type III/Sanfilippo (MIM 252900) patients have shown reduced excretion of HS and no serious adverse effects, albeit with limited clinical efficacy and benefit [45–47]. Another tool inhibitor for GAG chain elongation, rhodamine B, showed improvement for neurological and skeletal disease symptoms in a mouse model of MPS type I (MIM 252800) [48].

Considering genistein and rhodamine B are non-specific inhibitors, targeted inhibition of specific biosynthetic enzymes is desired. To this end, RNA interference (RNAi) technology has been used to silence GAG synthesis genes, encoding enzymes involved in the chain initiation steps that are common to all GAGs (e.g. XYLT1, XYLT2, GALTI, and GALTII [49]), as well as enzymes specific to HS chain elongation (e.g. EXTL2 and EXTL3 [50,51]). These studies together demonstrated decrease in the targeted mRNA concomitant with reduced GAG biosynthesis and attenuated phenotypes in MPS I and III patient fibroblasts. Crossing a mouse model of MPS type III with mice harbouring one null copy of *Ext1* or *Ext2*, also reduced HS and biomarker in multiple tissues [52].

#### Glycogen

Andersen disease (MIM 232500) and adult polyglucosan body disease (APBD; MIM 263570) caused by glycogen branching enzyme (GBE1) deficiency, as well as Lafora disease (MIM 254780) due to mutations in enzymes involved in glycogen dephosphorylation (malin and laforin), are neurological diseases sharing a common neuropathology of malformed glycogen (‘polyglucosan’) accumulation. Additionally, Pompe disease (MIM 232300), caused by deficient acid alpha-glucosidase (GAA) required for glycogen breakdown, leads to progressive lysosomal accumulation of glycogen in cardiac and skeletal muscle. While these storage disorders impact different aspects of glycogen metabolism (Figure 1C), down-regulation of glycogen synthesis to lessen the storage burden could represent a universal SRT approach, since the common hallmark among these disorders is muscle glycogen accumulation. Indeed, knockdown of the muscle glycogen synthase gene *GYS1* eliminated polyglucosan formation and restored neurological functions in a Lafora mouse model [53,54], as well as a neuronal APBD model [55]. ASO- or RNAi-mediated knockout of *GYS1* in Pompe mouse also significantly decreased aberrant accumulation of lysosomal glycogen [56,57]. Humans who have a total absence of GYS1 due to rare inherited mutations (MIM 611556) are healthy except for late-childhood cardiomyopathy, while no health issues were found with 50% residual GYS1 activities [58,59]. In line with the SRT approach, targeting the hepatic glycogen synthase isozyme GYS2 could provide rescue for those GSDs causing liver injuries. To this end, RNAi silencing of *GYS2* prevented glycogen accumulation in mouse model of GSD type III (MIM 232400), and reduced hepatic steatosis in mouse model of GSD type Ia (MIM 232200) [60], both being defects of glycogen breakdown.

#### Calcium oxalate

Primary hyperoxaluria type 1 (PH1, MIM 259900), due to deficiency in peroxisomal alanine–glyoxylate aminotransferase (AGXT; Figure 1D), results in the accumulation of glyoxylate that is converted to insoluble oxalate and deposited in the kidney, leading to kidney stone formation and ultimately end-stage renal disease. An SRT approach to inhibit glyoxylate biosynthetic enzymes, hence attenuating oxalate formation, could provide therapeutic benefit for PH1. One candidate target is glycolate oxidase (HAO1), the enzyme immediately upstream of defective AGXT in peroxisomal glyoxylate metabolism. A *HAO1*-deficient mouse [61] and individuals carrying rare *HAO1* mutations [62] are asymptomatic, while a double *AGXT/HAO1* knockout mouse demonstrated lower oxalate secretion [61]. siRNA targeting *HAO1* reduced urinary oxalate by 50% in PH1 models from mouse [63,64] and other primates [65]. Phase I clinical trial for this RNAi therapeutic is currently underway. Another contributor to glyoxylate production, mitochondrial hydroxyproline dehydrogenase (PRODH2) [66], also shows viability as a potential SRT target, based on siRNA knockdown in PH1 mice [64] and chemical inhibition in a Drosophila model [67]. More recently, RNAi-mediated knockdown of lactate dehydrogenase (LDH), proposed to be the culprit enzyme converting glyoxylate to oxalate, efficiently reduced oxalate crystal formation in PH1 mice [68]. These strategies have the potential to supplant the current mainstay treatment of combined kidney–liver transplantation.

### High-throughput screening approaches to identify new chemical starting points

The next step for novel validated therapeutic targets would often be entry into a drug discovery pipeline, with the aim of identifying hit compounds that would exert specific effects towards the target. To achieve this, conventional high-throughput screening (HTS) campaigns have often involved bespoke activity assays *in vitro* to interrogate the catalytic functions of the target protein, using a library of small molecule compounds. An example of HTS that has been applied to SRT is the screening for galactokinase (GALK1) inhibitors, an enzyme preceding galactose-1-phosphate uridylyltransferase (GALT) in galactose metabolism (Figure 1E). *GALT* mutations cause classic galactosaemia (MIM 230400), the pathological driver of which is partly attributed to the toxicity of accumulating substrate (galactose-1-phosphate, Gal-1-P) from defective GALT enzyme, hence the approach of inhibiting upstream GALK1 to attenuate Gal-1-P levels has been proposed as SRT [69]. A HTS campaign employing a luciferase-based activity assay was launched to screen recombinant GALK1 enzyme against 50K compounds initially [70], and later on a larger diverse set of ∼274 000 compounds [71], yielding hits from dihydropyrimidine and benzoxazole series with low-uM IC_50_, which reduced Gal-1-P concentrations in patient-derived cells [71,72].

Other medium/high-throughput screening efforts for SRT inhibitors have applied target-specific cell-based assays. In one study [73] for MPS, a reporter gene assay was used to screen 1200 compounds for transcriptional inhibition of NDST1, an enzyme involved in the early stage of HS synthesis. Because of its role, hits identified for this enzyme could have therapeutic potential for a subset of six MPS subtypes due to defective HS degradation. In another study [74] for PH1, genes involved in peroxisomal glyoxylate metabolism were stably transfected into CHO cells which lacked this pathway, to screen for compounds that rescued oxalate-induced cell death. This approach would potentially cover different modes of action, i.e. not only substrate reduction in upstream enzyme HAO1, but also direct/indirect rescue of the defective enzyme AGXT.

Structure-based drug design, which has facilitated greatly the development of small molecule therapeutics for many aspects of human medicine (for review, see [75]), has yet to contribute significantly towards the IEM field [76,77], although the emerging SRT targets would be ideally suited for structural characterization. Nowadays, technological advances have sufficiently reduced the timeframe of protein structure determination, to enable the application of X-ray crystallography as a screening method for drug discovery. To this end, fragment-based screening by crystallography has emerged as a tool to identify initial chemical starting points for a novel drug target, where there are no prior tool inhibitors available. This approach is taken in the authors' laboratory [78], to identify inhibitors of SRT targets such as HAO1 [79] for PH1.

### Beyond small molecules: genetic SRT holds promise

Gene silencing approaches, exploiting RNAi and ASO technologies, have revolutionized loss-of-gene functional analysis in the past decade. They have been used in POC studies to validate potential SRT targets, aimed at selectively down-regulating specific genes and evaluating the knockdown impact on mRNA and protein levels, as well as on the pathology associated with toxic substrate accumulation, in cell culture and animal studies. A few of these POC studies have already been described in the previous section. More recently, RNAi and ASO molecules are being developed into the next-generation therapeutics as an alternative modality to small molecules, which leads to the coining of the phrase genetic SRT [80,81]. The transformation of these genetic SRTs from mechanistic tools in the bench into approved therapeutics in the clinic has had to encounter many technological hurdles that include nucleotide delivery, stability, and immunogenicity.

RNAi exploits the endogenous machinery of post-transcriptional gene silencing, mediated by siRNA. The siRNA (synthetic or vector-based) is double-stranded, where one strand is complementary to the target mRNA, and the other strand engages the RNA interference silencing complex (RISC) and recruits it for sequence-specific cleavage of the target mRNA. Significant inroads have been made to improve RNAi delivery into target cells, thereby evading nuclease degradation, accumulation into endosome, and triggering of immune response (for review, see [82]). Some innovative solutions include encapsulating the siRNA in a lipid nanoparticle [83] or dynamic polyconjugates [84], as well as tethering siRNA to *N*-acetylgalactosamine (GalNAc) for targeting to the liver [85]. The first marketed RNAi therapeutic, approved in 2018, is Patisiran (Onpattro, Alnylam), administered for the rare misfolding disorder hereditary transthyretin (TTR) amyloidosis [86], where liver-derived amyloidic mutant TTR protein leads to multi-organ dysfunction. Some of the novel RNAi therapeutics in the clinical development pipeline for SRT, all targeting liver diseases, include: ALN-AS1 (Givosiran) silencing the haem biosynthesis gene *ALAS1* as treatment for acute intermittent porphyria (MIM 176000), currently under Phase III trial [87]; and ALN-GO1 (Lumasiran) silencing *HAO1*, having completed Phase II [65], as well as DCR-PHXC (Phase I) silencing the *LDH* gene [68], as treatment for PH1.

In contrast with RNAi, ASOs are single-stranded DNA that bind directly to the target mRNA, to silence gene expression by engaging cellular RNase H for mRNA degradation, or steric blocking of protein translation machinery, or exon skipping. Like RNAi, advances in the delivery technology such as GalNAc conjugation have improved ASO potency significantly [88]. Several ASO drugs have already been approved by the FDA for clinical use (see [89] for summary). To the best of our knowledge, the only ASO application to SRT, to date, is the aforementioned pre-clinical study of suppressing muscle *GYS1* expression for Pompe [56]. Here, antisense is accomplished by means of a phosphorodiamidate morpholino oligonucleotide, which invokes exon skipping in *GYS1*, conjugated to a cell penetrating peptide for delivery into the Pompe mice.

## Concluding remarks

With the marketed approval of existing SRT drugs already over a decade ago, the emergence of the next SRT therapeutics, be they small molecules or RNAi, is long due. Considering the effectiveness of SRT could somewhat depend on the degree of residual activity in the defective enzyme, the years to come will probably see the application of SRT not only as monotherapy but also in combination with adjunct approaches aimed at enhancing the defective enzyme, such as ERT and PC (for review, see [81]). Expanding beyond the arena of IEMs, SRT could potentially reveal new indications for many common diseases of cancer [90], endocrine [91], and neural [92] biology, in which abnormal metabolite level is integral to their disease pathology.

## Summary

- SRT addresses the disease pathology associated with toxic accumulation of metabolites, by down-regulating the enzymatic step(s) involved in the biosynthesis of the metabolites.
- The marketed approval of SRT for tyrosinaemia and GD over a decade ago has paved the way for the application of this approach to other IEM with unmet medical need.
- With the advent of gene silencing and high-throughput compound screening technologies, novel inhibitory targets and novel chemical modalities will emerge as the next-generation SRT.

## Abbreviations

AGXT: alanine–glyoxylate aminotransferase
APBD: adult polyglucosan body disease
ASO: antisense oligonucleotide
BBB: blood–brain barrier
ERT: enzyme replacement therapy
FAH: fumarylacetoacetate hydrolase
GAGs: glycosaminoglycans
GALK: galactokinase
GCS: glucosylceramide synthase
GD: Gaucher's disease
GSDs: glycogen storage disorders
GSL: glycosphingolipids
HS: heparan sulfate
HTS: high-throughput screening
IEM: inborn errors of metabolism
LDH: lactate dehydrogenase
LOF: loss-of-function
LSDs: lysosomal storage disorders
MPS: mucopolysaccharidoses
NPC: Niemann–Pick disease type C
PAH: phenylalanine hydroxylase
PC: pharmacological chaperoning
PH1: hyperoxaluria type 1
PKU: phenylketonuria
POC: proof-of-concept
RNAi: RNA interference
siRNA: small interference RNA
SRT: substrate reduction therapy
TTR: transthyretin
